# Supplementary material for: Assessment of clinical medical education needs inform design of a preceptor development program in Jordan: A multi method study
Source: PLoS One. 2025 Nov 19;20(11):e0337101. doi: 10.1371/journal.pone.0337101 (PMC12629491; doi:10.1371/journal.pone.0337101)
Supplement: S1 File — (DOCX) [file pone.0337101.s001.docx]

Training Need Assessment for Clinical Preceptors

* Required

1. This questionnaire will help us to structure a training course for the clinical educators according to their needs. You will be asked to choose your needs and to prioritize them. Your participation is

voluntary and anonymous. you can withdraw at any point during answering this survey. If you agree to participate, please choose agree and go to

the next question. Do you agree to participate in this Survey *


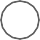

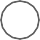
 Yes No

1. Kindly choose your training needs from the list below by classifying the workshop subjects from high priority to not a priority. **Make sure to distribute subjects on all categories** *

Not a

priority Low priority

Medium

priority High priority


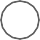

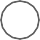

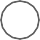

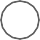

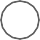

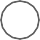

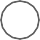

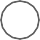
Lecturing for large groups Tutorial for small groups


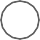

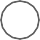

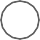

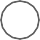
Bedside teaching (learning in the clinical setting)


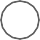

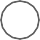

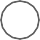

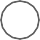

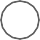

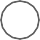

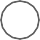

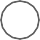

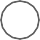

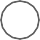

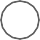

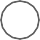

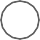

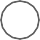

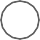

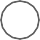
Providing feedback Assessment in clinical setting Clinical simulation Mentoring skills

1. Which of the following would influence you to register for preceptor development program (you can choose more than one option) *


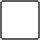
 Objectives
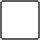
 Location
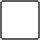
 Presenter


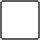
 Session length


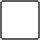

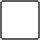
 Time of the session
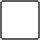
 CPD points

Certification

1. Please select the most desirable day(s) for you to attend training program (you can choose more than one option) *


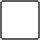
 Sunday
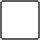
 Monday
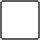
 Tuesday


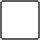

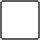
 Wednesday
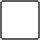
 Thursday

Saturday

1. Please select the most convenient time for you to attend preceptor development program (you can choose more than one option) *


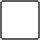
 8:00 am -10:00 am


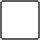
 10:00 am -12:00 noon


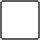
 12:00 noon-2:00 pm


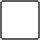

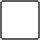
 2:00 pm-4:00 pm 4:00-pm-6:00 pm

1. Which method of professional development would you prefer the most *


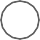
 Online


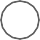
 Face-to-face


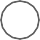
Blended (both online and face-to-face)

1. Kindly indicate your gender *


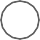

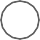
 Woman Man

1. Kindly indicate your specialty according to core board certification *


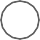
 Internal Medicine
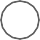
 Pediatrics


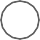

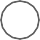
 Obstetric s and gynecology
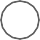
 Surgery

Other (Please specify below)

1. Your specialty is
2. Kindly indicate your years of expertise as a clinical educator *


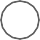
 1-5


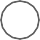
 6-10


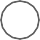
 11-15


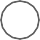
>15

1. Kindly indicate the health sector you currently work at *


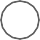
 University


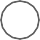

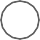
 Ministry of health
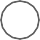
 Military services

Private sector

1. Please provide us with any comment that you believe will improve the intended training program

This content is neither created nor endorsed by Microsoft. The data you submit will be sent to the form owner.


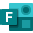
Microsoft Forms
