# Supplementary material for: Assessment of clinical medical education needs inform design of a preceptor development program in Jordan: A multi method study
Source: PLoS One. 2025 Nov 19;20(11):e0337101. doi: 10.1371/journal.pone.0337101 (PMC12629491; doi:10.1371/journal.pone.0337101)
Supplement: Attachment — Submitted filename: Response to Reviewers comments.docx [file pone.0337101.s004.docx]

Reviewers' comments:

Reviewer's Responses to Questions

**Comments to the Author**

1. Is the manuscript technically sound, and do the data support the conclusions?

The manuscript must describe a technically sound piece of scientific research with data that supports the conclusions. Experiments must have been conducted rigorously, with appropriate controls, replication, and sample sizes. The conclusions must be drawn appropriately based on the data presented.

Reviewer #1: Yes

Reviewer #2: Partly

2. Has the statistical analysis been performed appropriately and rigorously?

Reviewer #1: N/A

Reviewer #2: N/A

3. Have the authors made all data underlying the findings in their manuscript fully available?

The [PLOS Data policy](http://track.editorialmanager.com/CL0/http:%2F%2Fwww.plosone.org%2Fstatic%2Fpolicies.action%23sharing/1/010f019833f02bb6-aeccec9a-9d22-4211-b14f-e61190e783f5-000000/zEeJ-brkIB2pkZ-3SxQTUoCb1k_CKL3gRrBTXPvUN2A=220) requires authors to make all data underlying the findings described in their manuscript fully available without restriction, with rare exception (please refer to the Data Availability Statement in the manuscript PDF file). The data should be provided as part of the manuscript or its supporting information, or deposited to a public repository. For example, in addition to summary statistics, the data points behind means, medians and variance measures should be available. If there are restrictions on publicly sharing data—e.g. participant privacy or use of data from a third party—those must be specified.

Reviewer #1: Yes

Reviewer #2: No

4. Is the manuscript presented in an intelligible fashion and written in standard English?

PLOS ONE does not copyedit accepted manuscripts, so the language in submitted articles must be clear, correct, and unambiguous. Any typographical or grammatical errors should be corrected at revision, so please note any specific errors here.

Reviewer #1: Yes

Reviewer #2: Yes

5. Review Comments to the Author

Please use the space provided to explain your answers to the questions above. You may also include additional comments for the author, including concerns about dual publication, research ethics, or publication ethics. (Please upload your review as an attachment if it exceeds 20,000 characters)

**Reviewer #1:** This paper provides insights into variables that may impact clinical preceptor effectiveness in teaching. While limited to one country, the study had the advantage of studying six medical schools with significant numbers of preceptor and student participants to make the conclusions justified. I did not see that a copy of the written survey was readily available and readers may find this to be a useful appendix if they desire to investigate their own institution. Perhaps it was included and I somehow glossed over it in my reading of the manuscript.
The term 'clinical simulation' is intuitively obvious but I think a few sentences to describe what they mean might be helpful as a guide to this training need priority.
Table 3 outlining preferences for preceptor day and time for participation takes up a lot of space and could be reduced to a few sentences. The findings are an important variable but may likely differ among institutions/countries.

Reply: thank you for your valuable comments. The Questionnaire and the interview topics outlines had been already submitted as appendix files.

Clinical simulation: means the need to be trained how to deploy simulation scenarios. Clarified in lines: 162-163

Table 3 is omitted and the most important findings has been already mentioned in lines: 238-242

**Reviewer #2:** Overall, this paper represents an important attempt to define the gaps in clinical teaching in medical schools in Jordan, to inform preceptor training. The study design is appropriate, with use of both qualitative data from a survey instrument, and focus groups. The sample size is impressive.
The paper would benefit from some reorganization, so that the study part is clearly separated from the resulting curriculum development part. The analysis is limited to descriptive frequencies, which seems like a missed opportunity to me. With the range of respondents representing students, as well as faculty in two different settings, surely some meaningful comparisons could have been made. Did all the groups agree in their responses? I imagine that analysis of the student responses in the focus groups would yield some important differences from the responses of the faculty. If the focus groups were mixed, with students and preceptors together (which needs clarification), then would your participant coding permit any analysis by type of participant? I also think the work setting of the preceptors might have led to some differences in identified challenges and needs addressed in both the survey and the focus groups. With 400 survey respondents, you should be able to perform some analysis of respondent differences by health sector.

Author reply: Thank you for this valuable comment, though comparative analysis won’t significantly affect the design to syllabus, we compared the responses regarding training needs priority between the preceptors from university hospitals versus community preceptors.

Another problem for me as a reader is lack of familiarity with the Jordanian medical education system. An introduction of the system would be helpful to readers who may make assumptions based on the system they know. This could perhaps be provided in a graphical timeline format.

Author reply: Thank you for forwarding attention to this. A paragraph describing the Jordanian medical education system is added into the introduction.

In Jordan, medical education is provided over 6 years, the first 3 years includes basic sciences followed by three years of clinical sciences in the hospitals and health care centers from different sectors, universities, ministry of health and military medical services. It involves attending clinics, operation theatres, emergency rooms in addition to in patient wards. Clinical teaching is provided by academic staff from the faculties in addition to community preceptors from ministry of health and military medical services.

I will now provide detailed line-by-line comments and suggestions.
1. The word “Based” in the title is unnecessary, and the flow suggests that the assessment may also include assessment of the design of the curriculum. I recommend changing the title to: “Assessment of Clinical Medical Education Needs to Inform Design of a Preceptor Development Program in Jordan: A National Mixed Method Study.”

Thank you for your comment. The title changed as requested

2. The funding details statement appears to be missing some important required details.

Thank you for this comment. Details about the funder’s role is added.

3. The data availability statement does not appear to meet the PLOS One requirements.

Thank you for your comment. Data is now provided as supplementary file.

4. The abstract appears in two versions, the first starting on Page 2, line 32 and ending on page 3, line 58, and the second version starting on page 3, line 59 and ending on page 4, line 79. Even the key words are different. Please decide which abstract content you want to keep as your final.

Thank you for this comment. The first one is omitted.

5. Page 3, lines 61-63 in Background: “In this study,…” could be reworded for clarity to: “In this study, we explored the challenges faced by preceptors and students, and measured the educational needs of preceptors, to inform the design of a syllabus for a preceptor development program.”

Thank you for this comment. Changed as requested.

6. Page 4, line 65, the words “questionnaire” and “survey” are redundant. Please choose one.

Thank you for this comment. The word survey is deleted.

7. The introduction needs an overview of the medical education system in Jordan, including the time spent by students (do students enter medical school after college, or do the early years of medical school serve as college? Is the education divided into preclinical (classroom) training and then clinical training? Do the 6 years of medical school education include internship/residency? Are there required rotations and electives? Who are the preceptors? There is mention of some being community and some being academic and some Ministry of Health. In my country, medical students in their 3rd and 4th year have required inpatient rotations, mostly at the main teaching hospital, but also have outpatient rotations (some with academic faculty and some with community preceptors), and can take rotations at community hospitals. As you may imagine, the teaching skills and approaches vary immensely. This paper needs a better description of the preceptors, their sites/settings and their representation as teachers of the students. (If most of the precepting is done by community preceptors but most of your respondents were academic faculty, this would be an important weakness.)
8. On page 6 and 7, study design, I would like to see more granularity about the participants and their work settings. On lines 124-126, you state that “The study included clinical preceptors (academic faculty and Ministry of Health hospital staff)…” But elsewhere you refer to community preceptors (introduction, lines 83-86)? On page 7, lines 139-140, you refer to clinical instructors? Who are they? Are all the preceptors included in this study teaching in inpatient settings? Perhaps a graphic or at least a paragraph describing the preceptor types and their work settings is needed.

Thank you for this valuable comment.

9. Page 8 line 149, I think you mean “adapted” rather than “adopted”.

Thank you for this comment. Changed into adapted. The following paragraph is added into the introduction. In Jordan, medical education is provided over 6 years, the first 3 years includes basic sciences followed by three years of clinical sciences in the hospitals and health care centers from different sectors, universities, ministry of health and military medical services. It involves attending clinics, operation theatres, emergency rooms in addition to in patient wards. Clinical teaching is provided by academic staff from the faculties in addition to community preceptors from ministry of health and military medical services.

10. On page 8, line 153, I recommend clarifying that the prioritization used a Likert type scale (rather than a ranking). In the same line, it would improve reading comprehension to separate the training competency topics with semicolons.

Thank you for this comment. Prioritization involves choosing not a priority, low priority, medium priority, and high priority). This is added to the text. Semi colons are used as requested.

11. Page 8 line 161, I recommend change “among” to “during” and delete “up” In the date range.
Thank you for this comment. Changed as requested.

12. The dissemination should be better explained. How many preceptors were on the list? How many responded? Any response bias based on practice setting?

Thank you for this comment. The questionnaire was disseminated to group of preceptors and academics from each institute, each list included in average 100-125 Academic and preceptor. This is clarified in the manuscript

13. It would be helpful to know whether there were any research incentives for either the students or the preceptors.

Thank you for this query. There were no incentives for students or preceptors. it is added to the manuscript. Line 144

14. Page 9 Lines 166-171, I would like more information about the approach to thematic analysis. Did you use or adapt any specific approach to coding the focus group themes? If so, please explain and provide a reference.

Thank you for this valuable comment. Thematic analysis, as outlined by Braun and Clarke, was used to analyze the focus group transcripts. This approach involved a six-phase process: familiarization with the data, generation of initial codes, searching for themes, reviewing and refining themes, defining and naming themes, and producing the final analysis. This method enabled the identification of recurring patterns and key themes related to the training needs of clinical instructors. Braun, V., & Clarke, V. (2006). Using thematic analysis in psychology. Qualitative Research in Psychology, 3(2), 77–101. <https://doi.org/10.1191/1478088706qp063oa>

This clarification is added to the manuscript.

15. Page 9, lines 160-171, this sentence is confusing: “These themes made us aware of the modification of the questionnaire and the design of the training program syllabus needed.” Do you mean: “These themes informed needed revisions in the questionnaire and the eventual design of the training program syllabus.”?

Thank you for this comment. We meant that these themes were helpful in designing the program syllabus. The sentence is rephrased.

16. Page 9 lines 172-175 seems out of place and the section title is confusing. I think this belongs at the end (it is not really part of your study) as it describes the approach to designing the curriculum based on your study results. I recommend changing the header to “Syllabus Design Process”.

Thank you for this comment. The header changed as required.

17. The results section would benefit from more comparative analysis, as addressed in my introductory paragraphs.

Thank you for this valuable comment, we compared the responses regarding training needs priority between preceptors from University hospitals versus community hospitals’ preceptors (MOH, military, private).

18. On page 14, line 218, please clarify what you mean by “certification,” In my world, this has a very specific meaning, based on taking a board exam following specialty training. From context here, I believe you mean providing a training certificate.

Thank you for this comment. Actually training certificate what is meant. It is rephrased

19. Page 15, lines 226-227 – description of the eventual curriculum development does not belong in results.

Thank you for this comment. Actually it was improperly expressed, we meant the preliminary syllabus rather than curriculum, so it is rephrased into training preliminary syllabus.

20. Page 17, line 251, do you really mean that your training program would prepare preceptors to design simulation scenarios? This seems more ambitious than you can likely accomplish in the amount of time you have planned, and is likely beyond reach of many clinical preceptors. Perhaps you mean that they would be expected to use, adapt, deploy simulation scenarios that are provided to them?

Thank you for this valuable comment. The word Design is replaced with deploying.

21. Page 18, line 229, I recommend deleting the word “reflective”, which is redundant with “self-reflection a few words later.

Thank you for this comment. The word reflective is deleted.

22. Page 19, lines 19-282, I recommend rewording this to describe the study, designed to inform the curriculum, as the study is not really about the curriculum.

Thank you for this valuable comment. The study meant with the preliminary syllabus, therefore we rephrased the word curriculum into preliminary syllabus

23. I recommend reorganizing the discussion and shortening it. There is a great degree of redundancy. Discuss the results in reference to other related papers, then discuss how the results were applied in the planned curriculum.

Thank you for this comment. Discussion is changed as requested. Some repetitions were removed, our results were compared with another related paper, however no other similar English written papers can be found to compare our results with. We showed the significance of the identified core competencies and explained how it affected the content of the syllabus. In addition to showing the importance of the method used in our study.

24. There may be limitations based on selection bias of respondents, if the distribution of those responding did not reflect the range of preceptors. What is the risk that those who need the development course least are the most likely to take it (because they love to teach and are interested in it)?

Thank for this comment. Respondents were from different sectors and different specialties, to minimize the selection bias. However, the developed curriculum will be implemented into an obligatory course as a part of employment policy, it will be a prerequisite for all preceptors for either starting or continuing in the clinical education.

25. There are citations to several papers missing from the reference list: 16-19.

Thank you for this comment. References added to the reference list.

26. Page 22 Conclusion. You state: “By addressing challenges like over-enrollment and feedback deficiencies, the program aims to enhance medical education quality.” How does your study or your curriculum plan address the problem of over enrollment?

Thank you for this comment. Our curriculum doesn’t address the over-enrollment, we mean that our curriculum in addition to addressing the over enrollment both will enhance medical education equity. The word “by changed to after”.

6. PLOS authors have the option to publish the peer review history of their article ([what does this mean?](https://track.editorialmanager.com/CL0/https:%2F%2Fjournals.plos.org%2Fplosone%2Fs%2Feditorial-and-peer-review-process%23loc-peer-review-history/1/010f019833f02bb6-aeccec9a-9d22-4211-b14f-e61190e783f5-000000/fz-Bkma_jUEaYamTnogYo6i7FY4lJDFzPh14d18TNQQ=220)). If published, this will include your full peer review and any attached files.
