# Supplementary material for: Assessment of clinical medical education needs inform design of a preceptor development program in Jordan: A multi method study
Source: PLoS One. 2025 Nov 19;20(11):e0337101. doi: 10.1371/journal.pone.0337101 (PMC12629491; doi:10.1371/journal.pone.0337101)
Supplement: Attachment — Submitted filename: Rebuttal Letter.docx [file pone.0337101.s005.docx]

**Rebuttal Letter**

Dear Editor

We appreciate the valuable comments you provided us with. We addressed the comments as requested and we hope by this it fully fits the PLOS one’s publication criteria.

Kindly find below response to each comment.

Editor/ Reviewers Comments:

**Comment 1**: Please remove the word "national" in front of the mixed-method study.

Reply: thank you for your comment. The word national is removed and mixed changed into multi

**Comment 2**: Secondly, since the final outcome of the study is from an expert panel who developed the syllabus. The results of preliminary studies, focus group discussions and surveys were sequential, with the focus group discussion providing data used to contextualise an existing questionnaire for the subsequent phase. Thus, the study is best characterised by a sequential, multimethod design. have a look at these examples to guide you-<https://pubmed.ncbi.nlm.nih.gov/32554723/>,   <https://books.google.co.za/books?hl=en&lr=&id=F8BFOM8DCKoC&oi=fnd&pg=PA189&dq=sequential+multimethod+design&ots=gXgMyBoxJg&sig=l1bh8zh6jyYBJazHH3WuuL_m72I#v=onepage&q=sequential%20multimethod%20design&f=false>
Please rewrite the methodology session

Reply: thank you for your valuable comment, the method section is modified to a sequential multi method theme
